# Topological heterogeneity and evaporation dynamics of irregular water droplets

**DOI:** 10.1038/s41598-021-98115-4

**Published:** 2021-09-21

**Authors:** Yeseul Kim, Marta Gonçalves, Deok-Ho Kim, Byung Mook Weon

**Affiliations:** 1 Soft Matter Physics Laboratory, School of Advanced Materials Science and Engineering, SKKU Advanced Institute of Nanotechnology (SAINT), Sungkyunkwan University, Suwon, 16419 South Korea; 2 Department of Bioengineering, University of Washington, Seattle, WA 98195 USA; 3 Department of Biomedical Engineering, Johns Hopkins University, Baltimore, MD 21205 USA

**Keywords:** Soft materials, Fluids, Wetting

## Abstract

Water droplets sitting between wires are ubiquitous in nature and industry, often showing irregular (non-spherical) droplet shapes. To understand their topological singularity and evaporation mechanism, measuring volume changes of irregular water droplets is essential but highly challenging for small-volume water droplets. Here we experimentally explore topological heterogeneity and evaporation dynamics for irregular water droplets between wires with four-dimensional X-ray microtomography that directly provides images in three spatial dimensions as a function of time, enabling us to get three-dimensional structural and geometric information changes with time. We find that the topological heterogeneity of an irregular droplet is due to the local contact lines and the evaporation dynamics of an irregular droplet is governed by the effective contact radius. This study may offer an opportunity to understand how the topological heterogeneity contributes to the evaporation dynamics of irregular water droplets.

## Introduction

Small-volume ($<1 \upmu$L) water droplets sitting between wires are common in natural and industrial situations such as raindrops on spider wires and water droplets harvested on artificial wires^1–16^. To understand the wetting phenomena and the evaporation dynamics of irregular water droplets on wires, real-time and highly-precise visualization of small-volume water droplets is essential but difficult with conventional imaging techniques. The topological heterogeneity of the irregular droplet makes it challenging to acquire volume measurements unless one uses a precise and accurate mass balance, particularly for water droplets smaller than microliters. Visualizing the evaporation dynamics of irregular water droplets smaller than microliters and measuring their volume changes with time are highly challenging.

A tiny droplet on a flat solid substrate usually has topological homogeneity when the contact lines are identical at all radial directions and the droplet size is smaller than the capillary length. The capillary length is defined as $\lambda _{c} = \sqrt{\gamma /\rho g}$ (with the surface tension $\gamma$, the liquid density $\rho$, and the gravitational acceleration *g*), e.g., $\sim$2.7 mm for pure water. When the contact lines are not identical due to irregular contact points, the droplet may have topological heterogeneity because the contact lines are irregular. Despite diverse irregular droplets in nature and industry, their topological heterogeneity and evaporation dynamics are not clearly understood yet.

In this study, we experimentally explore the topological heterogeneity and the evaporation dynamics of irregular water droplets setting between crossing wires with four-dimensional (4D) X-ray microtomography that directly provides three-dimensional (3D) structural and geometric information that changes with time^17–19^. The high-speed high-resolution 4D X-ray microtomography enables us to achieve the 3D images changing with time for microliter or nanoliter irregular water droplets during evaporation. From our observations, we demonstrate how irregular water droplets evaporate in the air and show unprecedented evaporation dynamics for the irregular microliter water droplets. This study may offer an opportunity to understand the topological heterogeneity and the evaporation dynamics of irregular water droplets.

## Methods

To visualize evaporating irregular water droplets between two crossing glass wires, we utilized high-speed high-resolution 4D X-ray microtomography techniques^18^. We made two crossing glass wires from thin borosilicate glass tubes pulled by a commercial puller (Narishige PC-10, Japan). The thin glass tube was modified from the original tube (GC-1, Narishige Co., Japan) with an outer diameter and a thickness of 1.0 and 0.2 mm, respectively. The glass wires were fixed in a cross position and two distinct included angles at the crossing point were tested as 22.07$^{\circ }$ and 36.84$^{\circ }$. A dionized water (DI) droplet (0.1 μL and 0.3 μL) was gently placed on the junction between two crossing glass wires using a micropipette.

We used the 6C Biomedical Imaging (BMI) beamline in the Pohang Light Source II (PLS-II) that provided monochromatic synchrotron X-rays with 15 keV energy. The layout of the PLS-II 6C instrumentation included a scintillator (LuAG:Ce 50 μm), a 10X lens coupled with an sCMOS (PCO Dimax, PCO Imaging) camera, producing 2.22$\times$2.22 $\hbox {mm}^2$ projection images with 1.1 μm pixel resolution, taken from on-the-fly scanning within 180$^{\circ }$. The sample-to-scintillator distance was controlled as $\sim$100 mm. The exposure time for each projection was 5 ms and a total of 200 projection images for each microtomography set. The obtained projection images were processed with the OCTOPUS software (version 8.9.3, http://www.woorimtech.com/page/octopus411). After the reconstruction, the reconstructed images were loaded into Avizo software (version 2020.02, https://www.fei.com/software/avizo3d/). We applied non-local means filter to reduce the noise and improve image quality. Segmentation of background, water droplet, and wires was possible based on their relatively high attenuation coefficient difference, which depends on the material density and the atomic number. Thanks to the high spatial resolution of 1.1 μm and the high temporal resolution of 5 ms, the errors of segmentation were negligible. Based on the gray-scale intensity histogram, the threshold segmentation was carried out into the two different labels of droplets and wires. By measuring all the voxels in the droplet labels, we were able to measure the droplet volumes. By measuring the intersections between droplets and wires, we measured the contact surface areas. Finally, the reconstructed 3D images taken from X-ray microtomography of the same water droplet could be monitored as a function of time.

Additionally, the electronic balance (EX224G, Ohaus) was separately used to measure the evaporation dynamics of the irregular water droplets ($\sim$5 mg) on the two crossing glass capillaries. For comparison, the evaporation dynamics of a sessile droplet ($\sim$5 mg) on a 18$\times$18 mm^2^ cover glass substrate (Marienfeld) was also obtained. The droplet mass was measured every 1s and the mass-based experiments were repeated four times for each condition.

## Results and discussion

### Topological heterogeneity of irregular water droplets

**Figure 1:**
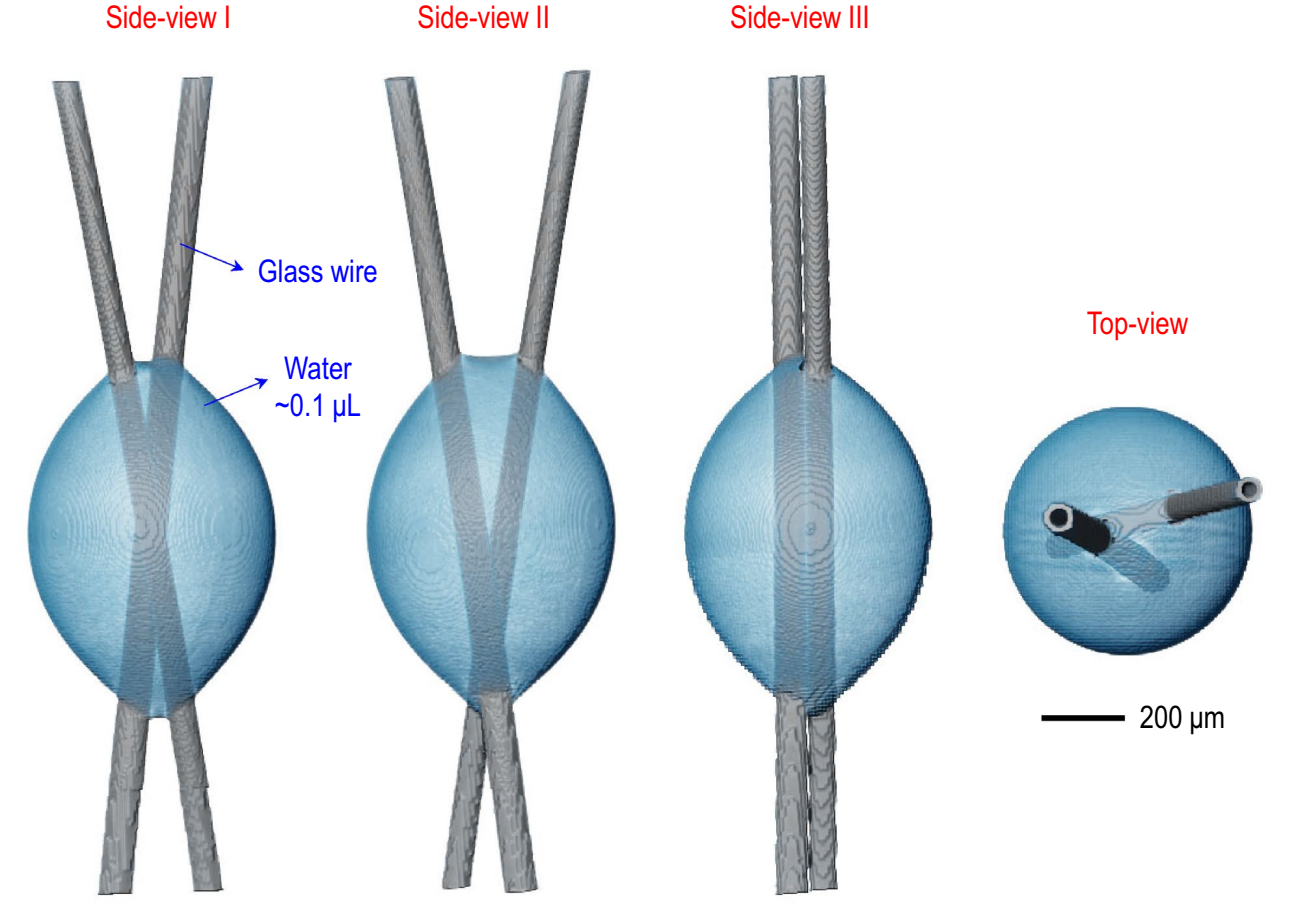
Topological heterogeneity of an irregular droplet. X-ray microtomography reveals the real shape of the initial 0.1 μL water droplet placed between two glass wires (where the crossing angle between wires is 22.07$^{\circ }$) at the initial time ($t = 0$). The microtomographic images are demonstrated at different angles (side-view I, II, III, and top-view). Despite that the droplet should be spherical by the surface tension, not by the gravity (because the droplet size is much smaller than the capillary length for water ($\sim$2.7 mm)), the droplet shape is deviated from the spherical cap, indicating the topological heterogeneity.

We first demonstrate the topological heterogeneity of the 0.1 μL irregular droplet placed between the crossing glass wires in Fig. 1. X-ray microtomography reveals the actual shape of the initial 0.1 μL water droplet placed between two glass wires at the initial time ($t = 0$). The X-ray microtomography images are illustrated at different angles (side-view I, II, III, and top-view). For tiny water droplets, particularly for the water droplet much smaller than the capillary length for pure water ($\sim$2.7 mm), we may suppose that the droplet should be spherical by the capillarity predominance. However, as proved by X-ray microtomography, the droplet shape is deviated from the spherical shape, indicating the topological heterogeneity of the irregular droplet.

The origin of the topological heterogeneity of the irregular water droplet would be the irregular contact lines of the water droplet. For instance, looking at the side-view I and II images in Fig. 1, the upper contact lines between two wires are not identical with the lower contact lines between wires, which induces the different contact lines between the upper and the lower triple (gas-liquid-solid) points. Every local water meniscus is always spherical between the solid (wire) surfaces due to the capillarity. In contrast, the whole water meniscus is not spherical eventually by the intermediate contacts on the wires. The spherical shape is seen from the top-view image without the intermediate contact lines in Fig. 1. This result suggests that the topological heterogeneity of the irregular droplet is governed by the capillarity and by the irregularity of the local contact lines.

### Evaporation dynamics of irregular water droplets

**Figure 2:**
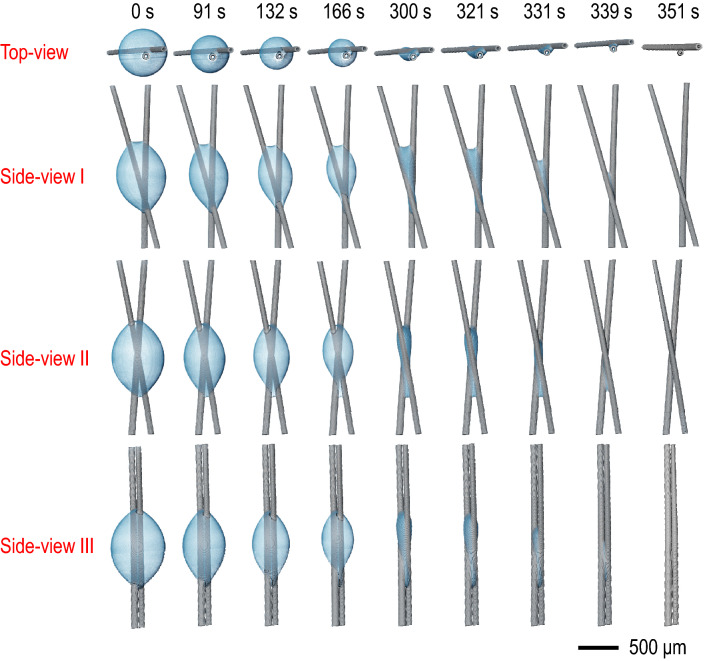
Topological heterogeneity during evaporation of an irregular droplet. Time evolution of the volume for an irregular water droplet between wires (where the crossing angle between wires is 22.07$^{\circ }$) was explored by 4D X-ray microtomography. The 3D microtomographic images taken for the different angles were taken with time, demonstrating the gradual volume decreases for the same irregular droplet with the topological heterogeneity.

We next consider the evaporation behavior of the 0.1 μL irregular droplet setting between the wires in Fig. 2, where the crossing angle between wires is 22.07$^{\circ }$. The time evolution of the volume for the irregular water droplet between wires is achieved by 4D X-ray microtomography. The microtomographic images taken for the different angles with the different evaporation times demonstrate the gradual volume decreases for the same irregular droplet despite the topological heterogeneity. During the evaporation process, the local spherical water meniscus maintains between the solid (wire) surfaces. In contrast, the whole water meniscus is not spherical by the presence of the intermediate contacts. The entire water volume gradually decreases with time.

**Figure 3:**
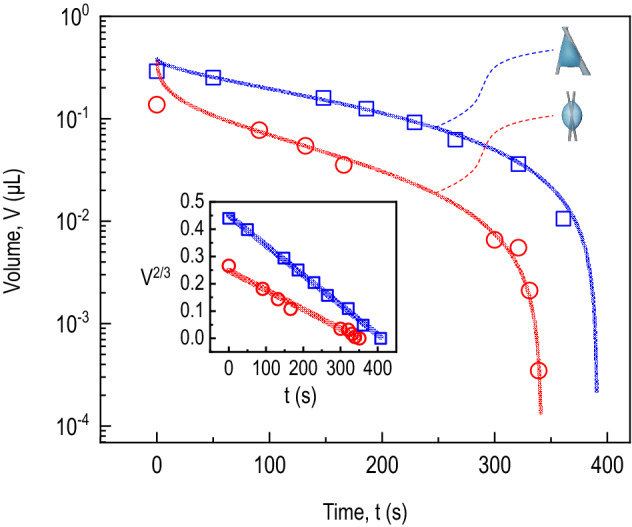
Evaporation dynamics of an irregular water droplet on glass wires. The droplet volume was measured by 4D X-ray microtomography for the initial 0.1 μL water droplet (circles: the crossing angle of the wires is 22.07$^{\circ }$, the same droplet in Fig. 2) and for the initial 0.3 μL water droplet (squares: the crossing angle of the wires is 36.84$^{\circ }$). The solid red and blue lines, taken from a power-law scaling, demonstrate that the droplet volume rapidly decreases with time. To understand the evaporation mechanism, we evaluate the volume$^{2/3}$ versus time (in the inset), demonstrating the linearity between V$^{2/3}$ and *t* (illustrated by the solid red and blue lines). This result suggests that the evaporation dynamics of an irregular droplet are governed mainly by the effective contact radius (equivalent to the contact surface area) induced by the topological heterogeneity.

To elucidate the evaporation dynamics of the 0.1 μL irregular droplet setting between the wires (as illustrated in Fig. 2, where the crossing angle between wires is 22.07$^{\circ }$), we measure the droplet volume taken by 4D X-ray microtomography in Fig. 3. For the initial 0.1 μL water droplet, we find a gradual decrease of the water volume. For guide, we first evaluate the water volume decrease with a power-law scaling as depicted by the solid red line as $V = V_{0} + \alpha t^{\beta }$ where $V_{0}$ is the initial volume and the parameters $\alpha$ and $\beta$ are the experimental constants. The evaporation rate $-dV/dt$ is given as $-\alpha \beta t^{\beta -1}$. We estimate $\alpha = -2.0472 \pm 24.2499$ and $\beta = 0.02422 \pm 0.25460$ (adj. $R^{2} = 0.98973$), implying $\alpha \beta \approx 0.05$ or an almost (not exactly) linear dependence on the evaporation time. We repeat the experiment with the initial 0.3 μL water droplet in Fig. 3, where the crossing angle between wires is 36.84$^{\circ }$. We estimate $\alpha = -0.01896 \pm 0.01236$ and $\beta = 0.50694 \pm 0.09115$ (adj. $R^{2} = 0.9938$), implying $\alpha \beta \approx 0.01$, as illustrated by the solid blue line in Fig. 3.

To understand the evaporation mechanism of the irregular water droplet, we evaluate the volume $$V^{2/3}$$ versus the evaporation time *t* (in the inset of Fig. 3), enabling us to achieve the linearity between $$V^{2/3}$$ and *t*. This $$V^{2/3}$$ versus *t* relation is natural in sessile spherical droplet evaporation^20^. The water volume changes at a given moment may be expressed as $$V^{2/3} = V_{0}^{2/3} - (2/3)k_{w}t$$ where $$k_{w}$$ is the evaporation rate coefficient that does not depend on time^20^. The evaporation rate is then determined as $$-dV/dt = k_{w}V^{1/3}$$. For the 0.1 μL irregular droplet (illustrated by the solid red line), we estimate $$V_{0}^{2/3} = 0.24767 \pm 0.00863$$ μ$$\hbox {L}^{2/3}$$ (corresponding to $$V_{0} \approx 0.1$$ μL) and $$(2/3)k_{w} = 7.10532\times 10^{-4} \pm 3.36077\times 10^{-5}$$ μ$$\hbox {L}^{2/3}$$/s (adj. $$R^{2} = 0.98238$$), indicating the evaporation rate $$-dV/dt \sim 0.5$$ nL/s for $$V_{0} \sim 0.1$$ $$\upmu $$L. For the 0.3 $$\upmu $$L irregular droplet (illustrated by the solid blue line), we estimate $$V_{0}^{2/3} = 0.4496 \pm 0.00469$$ $$\upmu $$$$\hbox {L}^{2/3}$$ (corresponding to $$V_{0} \approx 0.3$$ $$\upmu $$L) and $$(2/3)k_{w} = 0.00109 \pm 1.84548\times 10^{-5}$$ $$\upmu $$$$\hbox {L}^{2/3}$$/s (adj. $$R^{2} = 0.99771$$), indicating the evaporation rate $$-dV/dt \sim 0.7$$ nL/s for $$V_{0} \sim 0.3$$ $$\upmu $$L.

We discuss the spherical droplet model for the evaporation rates. The volume *V* and the surface area *S* of the droplet with the radius of the spherical droplet *r* are given as $V = \frac{4}{3}\pi r^{3}$ and $S = 4\pi r^{2}$. Assuming that the evaporation rate *dV*/*dt* is proportional to *S*, we obtain $-dV/dt = kS$ where *k* is the proportional constant. Therefore, $-dV/dt = k_{0}V^{2/3}$ with $k_{0} = 6^{2/3}\pi ^{1/3}k$. The $-dV/dt \propto V^{2/3}$ scaling is not valid in the inset of Fig. 3. This result suggests that for an irregular droplet system, the evaporation rate is not mainly dependent on the surface area, in contrast to a spherical droplet case.

**Figure 4:**
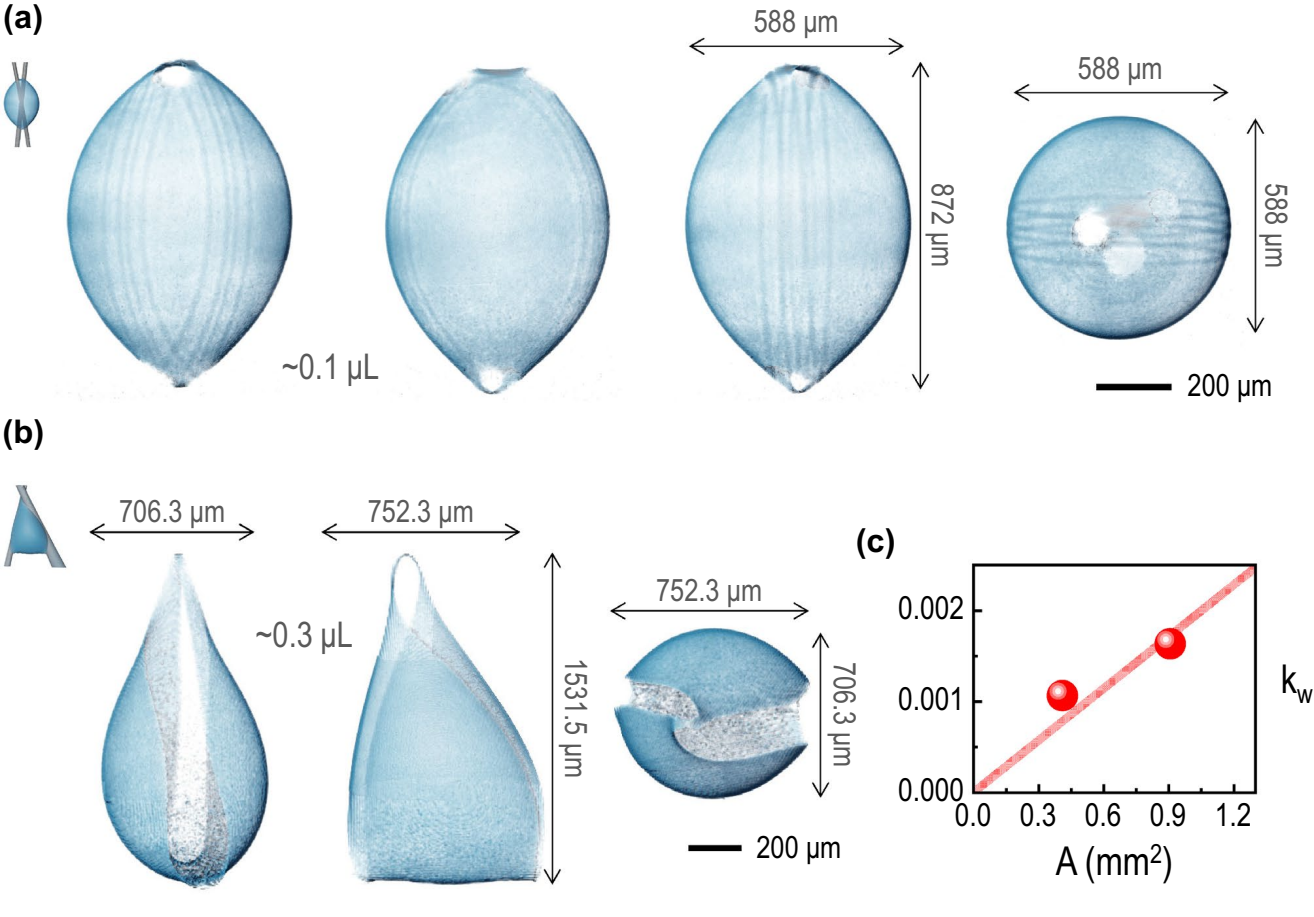
Two different irregular water droplets on glass wires, taken by 4D X-ray microtomography, (**a**) for the initial 0.1 $\upmu$L water droplet (the crossing angle of the wires is 22.07$^{\circ }$) and (**b**) for the initial 0.3 $\upmu$L water droplet (the crossing angle of the wires is 36.84$^{\circ }$). (**c**) The evaporation rate coefficients $k_{w}$ ($\propto -dV/dt$) [$\upmu$L$^{2/3}$/s] taken in the inset of Fig. 3 and the contact surface areas *A* [mm$^{2}$] measured by 4D X-ray microtomography, showing a proportionality, as described by the trend line as $k_{w} = 0.0019 A$ ($R^2 = 0.9767$).

We suppose that the evaporation rate would be proportional to the contact radius *R* of the irregular water droplet. The $-dV/dt \propto R$ ($\propto V^{1/3}$) scaling would be related to the vapor diffusion mechanism^20^. We estimate the effective contact radius as $R^{*} \sim 4R_{w} \sim 200$ $\upmu$m with the contact radius $R_{w} \sim 50$ $\upmu$m for the one-side water-wire contact point from Fig. 1. The measured evaporation rate of $\sim$ 0.5 nL/s at $V_{0} \sim 0.1$ nL in Fig. 3 is similar to the estimate of 0.5 nL/s for a 200-$\upmu$m-radius hemisphere sessile droplet, based on $-dm/dt = -\rho dV/dt$ ($\rho \approx 1000$ g/L is the water density) and the Hu-Larson evaporation equation^21^ as $-dm/dt = \pi RD (1 - H)c_{v} (0.27\theta ^{2} + 1.30)$ (where *D* is the vapor diffusion coefficient ($2.61\times 10^{-5}$ $\hbox {m}^{2}$/s), *H* the relative humidity, $c_{v}$ the saturated water vapor concentration (23.2 g/$\hbox {m}^{3}$), and $\theta$ the contact angle (1.57 radian for a hemisphere)) calculated by adopting normal conditions for water at room temperature and 20% relative humidity^20^. The effective contact radius is equivalent to the effective contact line and proportional to the contact surface area, which would control the evaporation rate of droplets of any shape^22^. From the X-ray microtomographic images of the initial 0.1 $\upmu$L droplet [Fig. 4a] and the initial 0.3 $\upmu$L droplet [Fig. 4b], we measure the contact surface areas. As illustrated in Fig. 4c, the evaporation rate coefficients ($k_{w}$ $\propto -dV/dt$) taken in the inset of Fig. 3 and the contact surface areas (*A*) measured by 4D X-ray microtomography show a proportionality, as described by $k_{w} = 0.0019 A$ ($R^2 = 0.9767$). This result suggests that the evaporation dynamics of the irregular droplet would be governed mainly by the effective contact radius and the diffusion-limited evaporation mechanism^20^, indicating that the water vapor flux would rapidly diverge at the contact lines as a kind of the edge effects^23^.

**Figure 5:**
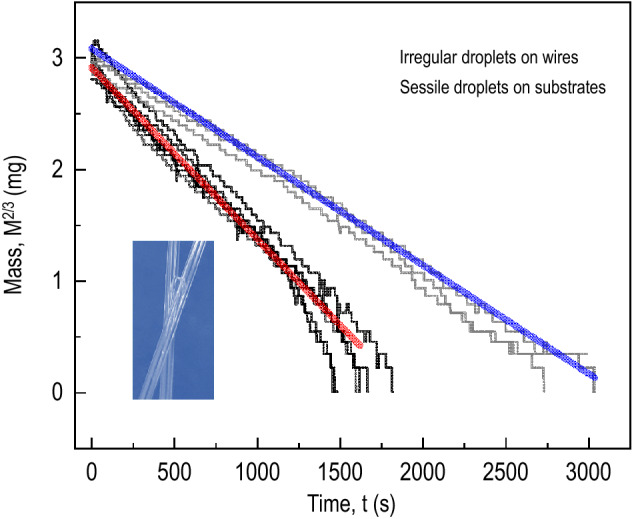
Evaporation dynamics of irregular water droplets on glass wires and sessile water droplets on flat glass substrates. The mass$^{2/3}$ versus time was taken by an electronic mass balance for the initial 5 mg water droplets, and the straightness, as demonstrated by the bold blue and red lines, was taken by the linear fits from the representative data curves. Interestingly, the evaporation rates of the irregular droplets are faster than the sessile droplets droplets, probably due to the effective contact line increases by the topological heterogeneity (see the inset).

The effective contact radius dependence of the evaporation rate or the $-dV/dt \propto R^{*}$ scaling is reproduced in a comparative study. We compare the evaporation dynamics of the irregular water droplets on the crossing capillary wires and the sessile water droplets on the flat substrates. The $\hbox {mass}^{2/3}$ ($\propto$ $\hbox {volume}^{2/3}$) versus time, taken by an electronic mass balance for the initial $\sim$5 mg water droplets, is illustrated in Fig. 5, showing the linearity of the evaporation dynamics. Interestingly, the irregular droplets evaporate faster than the sessile droplets, probably due to the topological heterogeneity that would increase the effective contact lines (and equivalently the contact surface areas). Further experiments can be carried out to test the effect of the intermolecular forces inside the small-volume water droplets on the evaporation dynamics. At the present work, we show that the evaporation rates of the irregular small-volume ($<1 \upmu$L) water droplets sitting between wires are dependent on the contact surface areas ($-dV/dt \propto k_{w} \propto R^{*} \propto A$).

## Conclusion

We experimentally explore how the irregular small-volume water droplet evaporates. The topological heterogeneity of the irregular water droplet is essential in understanding the actual wettability and the evaporation behaviors of the irregular water droplet. To measure the volume changes, we use the 4D X-ray microtomography that directly provides three-dimensional structural and geometric information as a function of time for the single irregular water droplet. We find that the evaporation dynamics of the irregular droplet is governed mainly by the effective contact lines. This study taken with the 4D X-ray microtomography guides a feasible way to characterize the topological complexity of the small-volume irregular water droplets that would determine the irregular contact lines, which provides a better understanding of the evaporation of the ultrasmall irregular water droplets.

## References

[1] Zheng Y (2010). Directional water collection on wetted spider silk. Nature.

[2] Ju J (2012). A multi-structural and multi-functional integrated fog collection system in cactus. Nat. Commun..

[3] Duprat C, Protiere S, Beebe A, Stone HA (2012). Wetting of flexible fibre arrays. Nature.

[4] Mei M, Fan J, Shou D (2013). The gravitational effect on the geometric profiles of droplets on horizontal fibers. Soft Matter.

[5] Weyer F, Lismont M, Dreesen L, Vandewalle N (2015). Compound droplet manipulations on fiber arrays. Soft Matter.

[6] Tadrist L, Motte L, Rahli O, Tadrist L (2019). Characterization of interface properties of fluids by evaporation of a capillary bridge. R. Soc. Open Sci..

[7] Sáenz P, Sefiane K, Kim J, Matar O, Valluri P (2015). Evaporation of sessile drops: a three-dimensional approach. J. Fluid Mech..

[8] Assouline S, Narkis K (2017). Evaporation from soil containers with irregular shapes. Water Resour. Res..

[9] Pan Z (2016). The upside-down water collection system of syntrichia caninervis. Nat. Plants.

[10] Weyer F, Duchesne A, Vandewalle N (2017). Switching behavior of droplets crossing nodes on a fiber network. Sci. Rep..

[11] Pan Z, Weyer F, Pitt WG, Vandewalle N, Truscott TT (2018). Drop on a bent fibre. Soft Matter.

[12] Tu Y, Wang R, Zhang Y, Wang J (2018). Progress and expectation of atmospheric water harvesting. Joule.

[13] Holweger H, Jamali M, Tafreshi HV (2021). Centrifugal detachment of compound droplets from fibers. Langmuir.

[14] Ruiz-Gutiérrez É, Ledesma-Aguilar R (2020). Lattice-boltzmann simulations of the dynamics of liquid barrels. J. Phys.: Condens. Matter.

[15] Wang F, Schiller UD (2021). Hysteresis in spreading and retraction of liquid droplets on parallel fiber rails. Soft Matter.

[16] Wu R, Kim T (2021). Review of microfluidic approaches for fabricating intelligent fiber devices: importance of shape characteristics. Lab Chip.

[17] Jung JW (2014). Four-dimensional visualization of rising microbubbles. Sci. Rep..

[18] Park HK, Kim Y, Min H, Pang C, Weon BM (2019). Hexagonal deposits of colloidal particles. Phys. Rev. E.

[19] Kim Y, Lee S, Lim J, Weon BM (2020). X-ray nanotomography of dry colloidal packings. Sci. Rep..

[20] Cho K (2016). Low internal pressure in femtoliter water capillary bridges reduces evaporation rates. Sci. Rep..

[21] Hu H, Larson RG (2002). Evaporation of a sessile droplet on a substrate. J. Phys. Chem. B.

[22] Sazhin S (1994). Droplets and Sprays.

[23] Bocquet L (2007). Tasting edge effects. Am. J. Phys..

